# Fabrication and Characterization of Chitosan/Poly(Lactic-Co-glycolic Acid) Core-Shell Nanoparticles by Coaxial Electrospray Technology for Dual Delivery of Natamycin and Clotrimazole

**DOI:** 10.3389/fbioe.2021.635485

**Published:** 2021-03-05

**Authors:** Xiaoming Cui, Xiaoli Li, Zhilu Xu, Xiuwen Guan, Jinlong Ma, Dejun Ding, Weifen Zhang

**Affiliations:** ^1^College of Pharmacy, Weifang Medical University, Weifang, China; ^2^Department of Pharmacy, Weifang Traditional Chinese Hospital, Weifang, China; ^3^Collaborative Innovation Center for Target Drug Delivery System, Weifang Medical University, Weifang, China; ^4^Shandong Engineering Research Center for Smart Materials and Regenerative Medicine, Weifang Medical University, Weifang, China

**Keywords:** natamycin, clotrimazole, core-shell structure, coaxial electrospray, *C. albicans*

## Abstract

Natamycin (NAT) is the drug of choice for the treatment of fungal keratitis (FK). However, its inherent shortcomings, such as poor solubility, high dosing frequency, and long treatment cycle, need to be urgently addressed by designing a new delivery to widen its clinical utility. Growing research has confirmed that clotrimazole (CLZ) plays a significant role in fungal growth inhibition. Hence, coaxial electrospray (CO-ES) technology is used herein to prepare nano-systems with an average hydrodynamic particle size of 309-406 nm for the co-delivery of NAT and CLZ in chitosan (CTS) and poly(lactic-co-glycolic acid) (PLGA). The resulting NAT/CLZ@CTS/PLGA formulations were characterized by a transmission electron microscope (TEM) and *in vitro* release test. The results show that the formulations had obvious core-shell structures, uniform particle distribution, and also can sustain the release of drugs over 36 h. Furthermore, *in vitro* hemolysis, *in vivo* corneal irritation test, local allergenic test, and antifungal activity analyses are performed to evaluate the safety and efficiency of the formulations. Thus, good biosafety along with a significant anti-candidiasis effect are found in the NAT/CLZ@CTS/PLGA nanoparticles (NPs). Taken together, the results suggest that this design may provide a promising drug delivery system and a new option for the treatment of FK.

## Introduction

Fungal keratitis (FK) is a major form of inflammation that is accompanied by vision loss and blindness (Chodosh, 2011; Liu et al., 2013). Moreover, its relatively high prevalence makes it a very serious burden for health care systems around the tropical regions such as South India, Ghana, and China (Roy et al., 2019). The main fungal species, causing corneal fungal inflammation, are *Fusarium*, *Aspergillus*, and *Candida*, with *Candida* being one of the most important pathogenic fungi. Meanwhile, natamycin (NAT) is the only topical ophthalmic drug with powerful antifungal effects and few side effects that has been approved by the Food and Drug Administration (FDA) (Dietrich, 2010; Mimouni et al., 2014). The antifungal mechanism of NAT is to damage the permeability of the cell membrane by binding with ergosterol and to disrupt the normal metabolism and growth of the fungus. Despite its worldwide use in the treatment of FK, a long treatment time is incurred when NAT is used alone and, hence, adjuvant therapy with other drugs is usually required. In particular, clotrimazole (CLZ) kills the fungal cell by inhibiting the biosynthesis of triglycerides and phospholipids along with the activity of oxidase and peroxidase, making this a broad-spectrum antimycotic drug. In addition, CLZ also has the capability to interfere with the transformation of *Candida albicans* from the spore form to the invasive hyphae (Adams, 1990; Govender, 2015). Therefore, it has been hypothesized that the combination of NAT with CLZ might have a synergistic effect upon the treatment of FK. In practice, however, NAT and CLZ presented the disadvantages of low water solubility, rapid metabolism, and high dosing frequency, thus limiting their therapeutic potential. Hence, there is a need to improve the delivery form in order to address these problems (Chugh, 2014).

Following the rapid development of nanomaterials, many researches have focused on the use of nanomaterials-based drug delivery systems in order to increase the bioavailability, prolong residence time, and reduce the side effects of various drugs (Yamada, 2014). According to the existing literature, more and more materials have been extensively studied and successfully designed for topical ocular delivery, such as poly(glycerol sebacate) (Chegini et al., 2019), eudragit (Rathod et al., 2017), and gold (Masse et al., 2019). However, a major challenge with these nanomaterial-based drug delivery systems is that the materials have unknown or objectionable toxicity profiles and are not approved by the regulatory authorities.

Poly(lactic-co-glycolic acid) (PLGA) is one of the most successfully developed biodegradable polymers with good biocompatibility and low toxicity, which has been designed for use in implants, scaffolds, and nanoparticles (NPs) (Almería et al., 2010; Żywicka et al., 2017). Furthermore, chitosan (CTS), a natural polycationic linear polysaccharide with outstanding biocompatibility and antibacterial action, has been considered as a promising nanomaterial (Rozman et al., 2019). Hence, various biomedical and pharmaceutical CTS-based microns or NPs have been developed to enhance the therapeutic effect and reduce irritant or allergic reactions (Diebold, 2010; Gonçalves, 2017). These studies inspired the present authors to use CTS and PLGA in the development of a nano-vehicle system for the dual delivery of NAT and CLZ exclusively via FDA-approved carrier materials (Castro et al., 2019; Jiménez-López et al., 2019).

**FIGURE 1:**
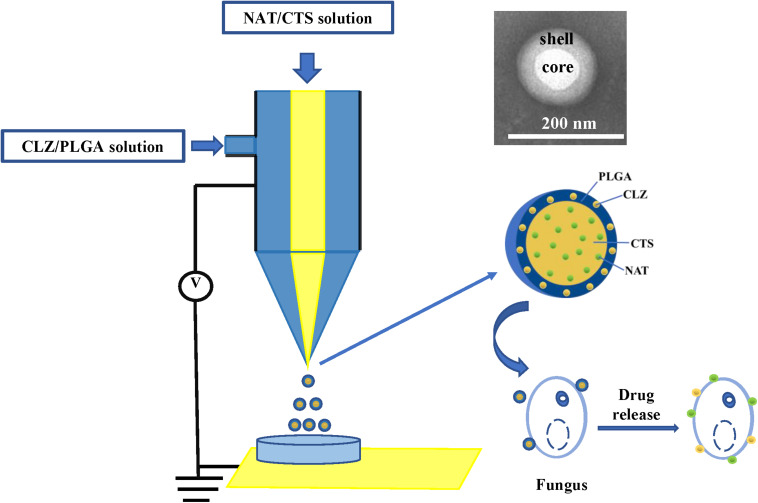
Schematic that shows the preparation and function of NAT/CLZ@CTS/PLGA NPs.

Presently, co-delivery of immiscible drugs via ocular administration to provide more efficiency remains challenging. Hence, new technology for immiscible dual-drug co-delivery is highly sought after. In this respect, coaxial electrospray (CO-ES) technology (Miller, 2017) is an emerging technology that was first reported by Loscertales (2002) as a method for generating steady coaxial jets of immiscible liquids and is now widely used in the preparation of core–shell micro-/nanoparticles (Soares et al., 2018). Some studies have reported that NPs prepared using CO-ES technology presented the better physicochemical features than those obtained using the common technologies due to the high Entrapment Efficiency (EE), simple preparation steps, large production scale, and fewer limitations of CO-ES with respect to the applied materials (Jaworek, 2007; Sridhar and Ramakrishna, 2013). In addition, CO-ES technology has displayed outstanding characteristics in the emerging biomedical and pharmaceutical fields, and is a powerful tool for the co-delivery of drugs (Xie et al., 2008; Rasekh et al., 2017). More recently, our group also developed core-shell magnetic NPs for the treatment of non-small cell lung cancer (NSCLC) and demonstrated that the core-shell drug delivery system has good characteristics and therapeutic effect (Yu H.L. et al., 2018). Herein, the CO-ES apparatus is used to generate a core-shell structure nano-system consisting of CTS- and PLGA-supported NAT and CLZ, for the synergistic treatment of FK therapy (Figure 1). In this study, the role of CLZ is to increase the therapeutic effect of NAT and shorten the FK treatment cycle. The carrier of this nano-system consists of CTS and PLGA, both of which exhibit acceptably good biocompatibility and biodegradability. Thus, a low-toxicity and high-efficiency system (designated as NAT/CLZ@CTS/PLGA NPs) is developed that can overcome the main limitations in application of NAT, including the high dosing frequency, low water solubility, and low efficiency, when used alone.

## Materials and Methods

### Materials

PLGA with a copolymer ratio of 50:50 and a molecular weight of 50,000 was obtained from Milan Biological Technology Co., Ltd (Dalian, China). CTS with a 96.1% degree of deacetylation (DAC) and a molecular weight of 12–16 KD was purchased from Hai Debei Marine Biotechnology Company (Jinan, China). NAT and CLZ were obtained from Sigma (St Louis, MO, United States). The Sabouraud glucose agar (SGA) medium and Sabouraud dextrose broth (SDB) were obtained from Shanghai Yuanye Bio-Technology Co. Ltd (Shanghai, China). All other reagents and solvents were analytical grade and used as received. The standard strain of *C. albicans* (Cat. No. ATCC^®^ MYA-5000^TM^) was procured from Beijing Zhongyuan Ltd (Beijing, China).

### Preparation of Electro-Sprayed NPs

The core-shell structured NAT/CLZ@CTS/PLGA NPs were fabricated following CO-ES technique. Briefly, PLGA was dissolved in acetone at a concentration of 1% (w/v) and then pumped it into the outer inlet of the coaxial nozzle at a feeding rate of 0.5 mm/min; a certain amount of CLZ was added into PLGA acetone solution. Furthermore, CTS was dissolved in 1% acetic acid at a concentration of 1% (w/v) and then pumped into the inner inlet of the coaxial nozzle at a feeding rate of 0.25 mm/min; a certain amount of NAT was added into the CTS acetic acid solution. The outer and inner diameters of the coxial nozzle were 0.8 mm and 0.5 mm, respectively, and the tip-to-collector distance was maintained at 10 cm. As the high positive voltage was 9.45 kV. The electro-sprayed NPs were collected by placing a glass Petri dish containing 10 ml of water, which was directly under the needle and then the formulations were dried under vacuum at room temperature.

Various NP formulations were produced, including the single drug-loaded formulations (NAT@CTS/PLGA and CLZ@CTS/PLGA) and the co-loaded formulation NAT/CLZ@CTS/PLGA.

### Morphology, Particle Size, and Zeta Potential

The morphology of the electro-sprayed NPs was visualized by transmission electron microscopy (TEM; Tecnai 20, FEI, United States). The particle size distribution (PSD) and Zeta potential value of different formulations were measured via dynamic light scattering (DLS) along with the data collection software provided with the Zetasizer Nano ZS90 (Malvern Zeta Potential/Particle Sizer Nano system, Malvern, United Kingdom). The experiments were carried out at 23°C and detected in three replicates.

### Drug Loading (DL) and Entrapment Efficiency (EE)

Drug analyses were performed via high-performance liquid chromatography (HPLC; Agilent 1260 infinity, Agilent Technologies, Palo Alto, CA, United States). The examined wavelengths were 221 nm for CLZ and 295 nm for NAT. In addition, the drug quantities were measured by calculating the amount of free drug in the filtrate. Briefly, 2-ml suspensions of electro-sprayed formulations were centrifuged at 12,000 rpm and 4°C for 30 min to separate the non-entrapped drugs from the suspensions. The supernatant was then filtered using a 0.22-μm microfilter membrane, and the drugs were analyzed via HPLC. The DL and EE values were then determined from Eqs. (1) and (2):

(1) $$\mathrm{DL}\left(\%\right)={\text{D}}_{1}/{\text{W}}_{\text{f}}\times 100\%$$

(2) $$\mathrm{EE}\left(\%\right)={\text{D}}_{1}/{\text{D}}_{\text{t}}\times 100\%$$

where D_*l*_ is the determined weight of drug in NPs, W_f_ is the weight of the drug-loaded NPs, and D_t_ is the weight of the total amount of drugs. All samples were performed at triplicate.

### Fourier Transform Infrared Spectroscopy (FT-IR)

The possible interactions between drug–excipient and excipient–excipient were determined by FT-IR (Avater-360, PerkinElmer, Waltham, MA, United States). The samples were prepared by mixing with potassium bromide (KBr) and then compressing. The spectra were collected in the range of wavenumbers from 4,000 to 500 cm^–1^.

### Thermogravimetric Analysis (TGA)

The physical properties of the samples were characterized by TGA (TG 201F1, Netzsch) in the temperature range of 10–800°C at a heating rate of 2°C/min under a flow of inert nitrogen (20 m^3^/s).

### X-Ray Diffraction (XRD)

The crystalline nature plays an important role in the stability, bioavailability, and solubility of a drug delivery system. Hence, the composition and crystalline structures of the samples were also characterized by XRD (X’Pert Power, Pan-alytical B.V., Netherlands) in the 2θ range of 5-90°.

### *In vitro* Drug Release

The *in vitro* release of NAT and CLZ from electro-sprayed NPs was performed using a dialysis bag (3,500 molecular weight cutoff, membrane-cell, Chicago, IL, United States) (Men et al., 2011). First, the samples were centrifuged at 12,000 rpm for 10 min and then the supernatant was discarded and the recovered NPs were rinsed with distilled water to exclude the free drugs. Subsequently, 2.0-ml samples were added into a dialysis bag, which was then immersed in PBS (50.0 ml) and gently swung at 37°C and 100 rpm. The release medium was sampled (1.0-ml) at predetermined time and immediately replaced with 1 ml fresh release medium (phosphate buffered saline, PBS, pH 7.4) after each sampling. The cumulative dose of NAT and CLZ was measured by HPLC method as described above.

### Hemolysis Assay

In the present study, the hemolysis assay was performed using a previously reported method (Yang et al., 2017). The fresh blood was obtained from New Zealand rabbit that was donated by the Pharmaceutical Laboratory of Weifang Medical University. Red blood cells (RBCs) were collected by centrifugation at 1,500 rpm for 20 min, followed by three washes with PBS. The RBCs (4% w/v) were then separately incubated in a 1:9 v/v ratio with various concentrations of the NAT@CTS/PLGA NPs, CLZ@CTS/PLGA NPs, and NAT/CLZ@CTS/PLGA NPs at 37°C for 3 h, followed by centrifugation at 12,000 rpm for 20 min. Then we measured the percentage of hemolysis by ultraviolet-visible (UV-vis) spectroscopy at 540 nm (Shimadzu UV-Vis Spectrophotometer UV-1700; Shimadzu, Kyoto, Japan). Distilled water was set as the positive control and PBS was set as the negative control. The hemolysis was calculated using Eq. (3):

(3) Hemolysis(%)=(As-An/(Ap-An)×100%

where A_s_ is the absorbance resulting from the addition of the NAT/CLZ@CTS/PLGA NPs to the erythrocyte suspension, A_n_ is the absorbance resulting from the addition of PBS to the erythrocyte suspension (negative control), and A_p_ is the absorbance resulting from the addition of distilled water to the erythrocyte suspension (positive control).

### Zone of Inhibition Tests

The antifungal activity of electro-sprayed NPs was assessed against *C. albicans* by the zone of inhibition (ZOI) test (Elbanna et al., 2014), which determined the level of antifungal activity that inhibits the growth of *C. albicans*. The NAT/CLZ@CTS/PLGA NPs were tested at a concentration of drugs equivalent to that of the NAT@CTS/PLGA NPs and CLZ@CTS/PLGA NPs. First, the *C. albicans* were co-cultured with sterilized SDB slants at 37°C and diluted with fresh medium to a concentration of 10^6^ CFU/ml after 24 h of co-culture. Meanwhile, the sterilized SGA medium was poured into a disposable Nest culture plate and allowed to coagulate at room temperature (25 ± 1°C). The *C. albicans* (0.5 ml) were then evenly swabbed onto each plate and a 6-mm-diameter sterilized filter paper was then placed in the center of each culture plate. Then, an 8-μl sample of the NAT/CLZ@CTS/PLGA NPs was added to a fresh, sterilized 6-mm-diameter filter paper in a separate plate, and PBS (0.02 M, pH 7.4) was added as the negative control. To allow sufficient time for the fungi to grow and the formulations to diffuse, all the plates were placed in a low-temperature incubator (Shanghai Jinghong Experimental Equipment Co. LTD, SHP-150, China) at 37°C for 48 h. The potential antifungal activity was then presented as the diameter (mm) of ZOI.

### Ocular Irritation Studies

For the *in vivo* ocular irritation studies, the irritancy and damaging effects of the NAT/CLZ@CTS/PLGA NPs and NAT/CLZ suspensions to the eyes were evaluated according to the modified Draize test (Bhatta et al., 2012). Each suspension (20 μl) was instilled directly into the cornea of the left eye of a rat at 30-min intervals for 6 h, while the cornea of a rat right eye was treated with PBS as a control. After treatment, observation was performed every 12 h for a total period of 72 h. To evaluate the level of eye irritation, the conjunctival congestion, secretions, and redness were each measured on a scale of 0 to 3, and the corneal opacity and swelling were each measured on a scale of 0 to 3. When the rats were sacrificed, the eyes were removed for hematoxylin and eosin (H&E) staining in order to visualize the microanatomy of the corneas.

### Local Allergenic Tests

In view of the safety of the NAT/CLZ@CTS/PLGA NPs, local allergenic test was performed on the skin of Sprague Dawley (SD) rats. A group of 10 7-week-old SD rats, half of which were male and half female, were obtained from Jinan Pengyue Experimental Animals Co., LTD. The rats were randomly divided into two groups (*n* = 5), namely, the NAT/CLZ suspension group and the NAT/CLZ@CTS/PLGA NPs group. Furthermore, the fur was then clipped from the backs of the rats and the test substance (NAT/CLZ suspension or NAT/CLZ@CTS/PLGA NPs, depending on the assigned group) was applied to the skin on the left-hand side at 1, 7, and 14 days and allowed to remain on the skin for 6 h. The control substance (PBS) was, similarly, applied to the skin on the right-hand side. After an additional 14 days, the treatments were applied to each group again and the rats were observed every 24 h for a total of 72 h. When the rats were sacrificed, the skin was taken out for H&E staining.

### Statistical Analysis

All experiments were performed in triplicates, and the obtained values were expressed as mean ± standard deviation (SD). All data were analyzed using the Statistical Package for the Social Sciences (SPSS) 19.0 (SPSS Inc., Chicago, IL, United States); the Student’s *t* test and one-way ANOVA were used to determine the statistical significance. A *p* value of < 0.05 was considered as statistically significant (Wang et al., 2018).

## Results and Discussion

### Particle Size, Zeta Potential, and Surface Morphology Measurement

**FIGURE 2:**
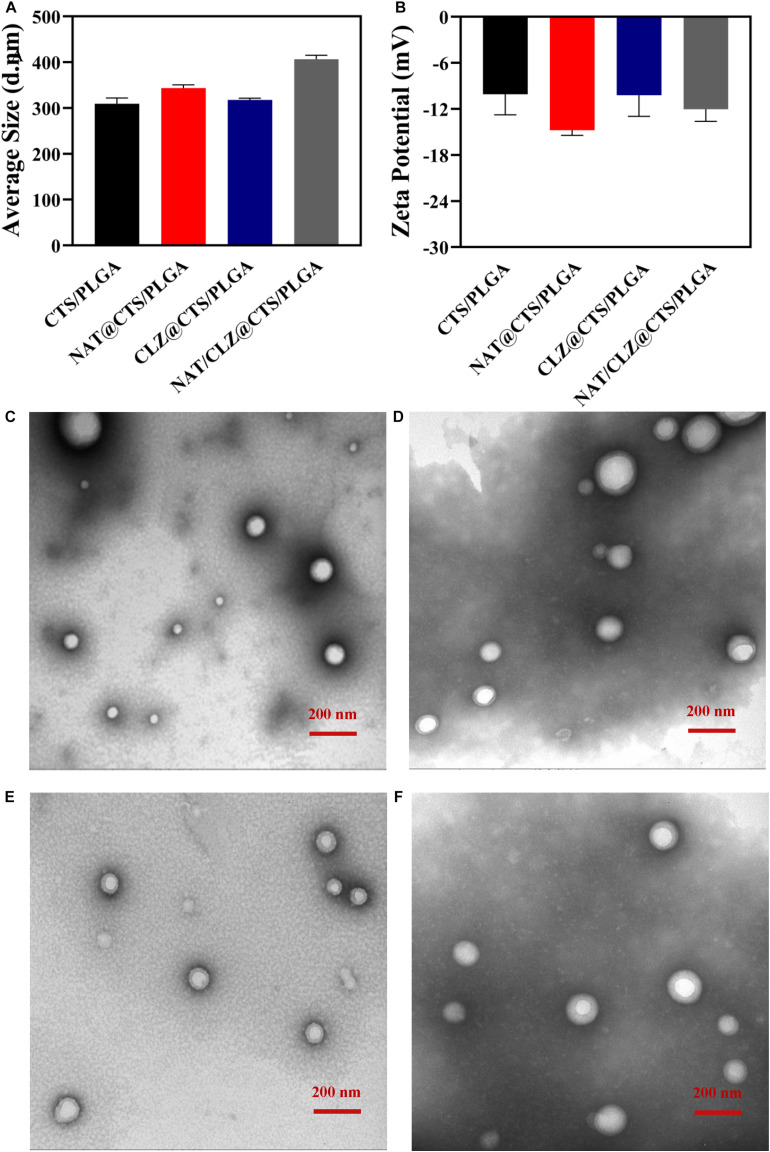
Structure of electro-sprayed NPs. **(A)** Histogram of hydrodynamic size. **(B)** Histogram of Zeta potential. **(C–F)** The morphology using TEM of **(C)** CTS/PLGA NPs, **(D)** NAT@CTS/PLGA NPs, **(E)** CLZ@CTS/PLGA NPs, and **(F)** NAT/CLZ@CTS/PLGA NPs.

The core-shell structured NAT/CLZ@CTS/PLGA NPs were fabricated following the CO-ES technique (Rasekh et al., 2017). The DLS measurements of the core-shell structured NAT/CLZ@CTS/PLGA NPs presented in Figures 2A,B indicate hydrodynamic diameters of 309–406 nm and zeta potentials ranging from -10 to -15 mV. In detail, the hydrodynamic diameters of the CTS/PLGA, the NAT@CTS/PLGA, the CLZ@CTS/PLGA, and the complete NAT/CLZ@CTS/PLGA NPs are 309.23 ± 12.76, 317.57 ± 7.32, 343.43 ± 3.70, and 406.63 ± 8.37 nm, respectively, while the corresponding zeta potentials are -10.08 ± 2.68, -14.7 ± 0.67, -10.21 ± 2.75, and -12.03 ± 1.58 mV, respectively. Thus, the presence of NAT and CLZ in the complete NAT/CLZ@CTS/PLGA formulation is indicated by a slight increase in the size of the NPs compared to the individual components (Table 1). while the corresponding components of the various formulations are shown in Table 1. Moreover, the presence of the NAT and CLZ in the NPs was slightly based on the increase in NP size. In addition, the representative TEM images of electro-sprayed NPs in Figures 2C–F clearly indicate the uniform size, spherical morphology, and smooth surfaces of the core-shell NPs. These results are reasonable and generally accepted for the application of NPs in drug delivery systems (Danhier et al., 2012; Zhou et al., 2018).

**TABLE 1:** Formulations of electro-sprayed NPs (mg).

**Formulation**	**PLGA**	**CTS**	**NAT**	**CLZ**
CTS/PLGA NPs a	50	25	–	–
NAT@CTS/PLGA NPs	50	25	5	–
CLZ@CTS/PLGA NPs	50	25	–	1
NAT/CLZ@CTS/PLGA NPs	50	25	5	1

### Drug Loading and Entrapment Efficiency

The drug-releasing characteristics of the various formulations are indicated by the percentage DL and EE values in Table 2. The results suggested that the NAT/CLZ@CTS/PLGA NPs (NAT) had an EE of 85.63 ± 0.04% and a DL of 6.65 ± 0.01%, and the NAT/CLZ@CTS/PLGA NPs (CLZ) have an EE of 89.61 ± 0.04% and a DL of 1.39 ± 0.01%. By comparison, the NAT (NAT@CTS/PLGA NPs) have EE and DL values of 79.29 ± 3.74% and 6.94 ± 0.32%, respectively, while the corresponding values for the CLZ (CLZ@CTS/PLGA NPs) are 88.22 ± 0.68% and 1.57 ± 0.01%, respectively. Moreover, the results of DL and EE in NAT/CLZ@CTS/PLGA NPs was very close to that in single drug-loaded NPs (NAT@CTS/PLGA NPs, CLZ@CTS/PLGA NPs). These results confirm that CO-ES technology can convey immiscible solution and successfully fabricate a co-loaded nano-based dual-drug delivery system with a high EE via a simple process. Compared with traditional methods, such as ionic cross-linking, emulsion method, and solvent evaporation technology, etc (EE of NAT, 40% to 73%) (Bhatta et al., 2012; Phan et al., 2014), CO-ES technology has unique capabilities such as excellent particle reproducibility, few restrictions on the materials, precise control over particle size, and high EE, even pack immiscible drugs (Chen et al., 2018; Dwivedi et al., 2018), presenting the potential to be a general method for preparing NPs.

**TABLE 2:** The results of drug loading and entrapment efficiency (mean ± SD, *n* = 3, %).

**Formulation**	**Drug loading**	**Entrapment efficiency**
NAT@CTS/PLGA NPs	6.94 ± 0.32	79.29 ± 3.74
CLZ@CTS/PLGA NPs	1.57 ± 0.01	88.22 ± 0.68
NAT/CLZ@CTS/PLGA NPs (NAT)	6.65 ± 0.01	85.63 ± 0.04
NAT/CLZ@CTS/PLGA NPs (CLZ)	1.39 ± 0.01	89.61 ± 0.04

### Fourier Transform Infrared Spectroscopy

**FIGURE 3:**
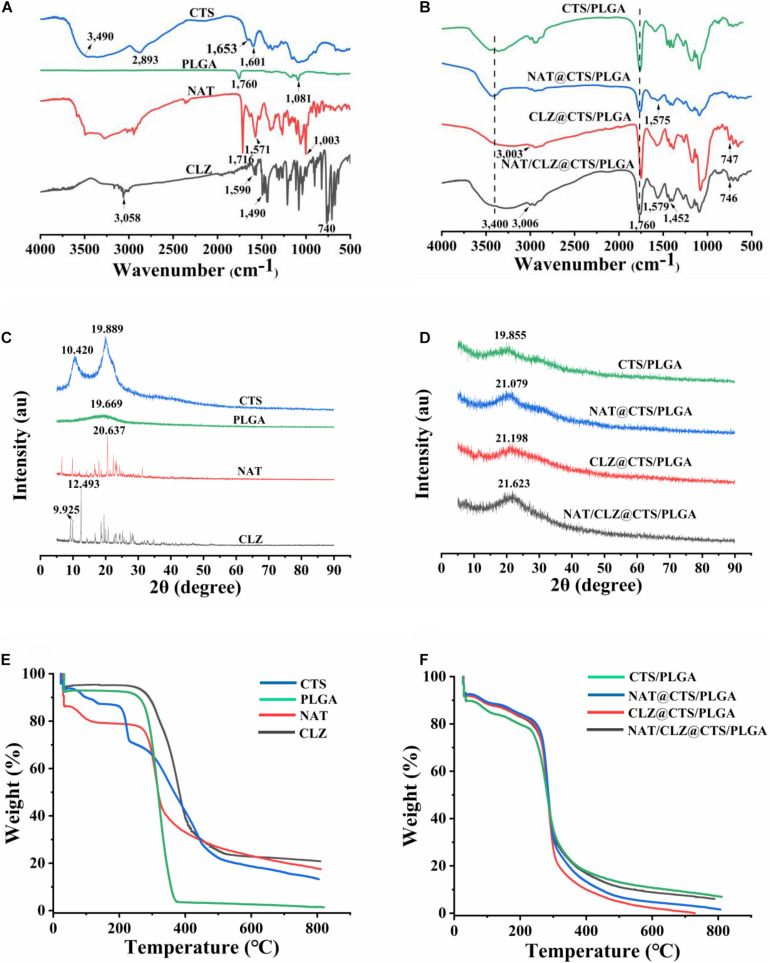
**(A)** FT-IR spectra of CTS, PLGA, NAT, and CLZ. **(B)** FT-IR spectra of electro-sprayed NPs. **(C)** XRD patterns of CTS, PLGA, NAT, and CLZ. **(D)** XRD patterns of electro-sprayed NPs. **(E)** TGA curves of CTS, PLGA, NAT, and CLZ. **(F)** TGA curves of electro-sprayed NPs.

To evaluate the possible drug–excipient and excipient–excipient chemical interactions, the FTIR spectra of the individual components and the electro-sprayed NPs are presented in Figures 3A,B, respectively. Thus, the FT-IR spectrum of the CTS exhibits many prominent peaks, including the following: the peak at 3,500–3,300 cm^–1^ involves -OH stretching, the peak at 1,653 cm^–1^ belongs to C = O stretching of the secondary amide (amide I band), and the peak at 1,601 cm^–1^ involves the N–H bending vibration (N-acetylated residues, amide II band) (Yu H. et al., 2018). Meanwhile, the PLGA exhibits the absorptions due to the ester group at 1,760 cm^–1^ and the C = O stretching at 1,081 cm^–1^ (Yang et al., 2019). The presence of NAT is confirmed by the characteristic adsorption band and peaks due to the conjugated ester absorption band, primary amine, and cyclic ether (Phan et al., 2014) of the copolymer at 1,716, 1,571, and 1,003 cm^–1^, respectively. In addition, the peaks observed at 3,058 and 740 cm^–1^ are attributed to the C–H aromatic stretch and C–H stretching of CLZ while the peaks at 1,590 and 1,490 cm^–1^ are related to the CLZ benzene ring stretching (Patel et al., 2019).

Meanwhile, the FTIR spectra of electro-sprayed NPs each exhibit outstanding absorption bands in the range of 3,500–3,300 cm^–1^, corresponding to the -OH group of CTS along with a peak (at ∼1,760 cm^–1^) associated with the C = O stretching of PLGA (Figure 3B). Moreover, compared with the spectrum of pure NAT, NAT-loaded electro-sprayed NPs all showed the characteristic peaks of NAT primary amine at appropriately 1,571 cm^–1^. Similarly, the CLZ-loaded electro-sprayed NPs all presented the characteristic peaks at appropriate ∼3,000 cm^–^1, related to the C–H aromatic stretch of CLZ. Moreover, no new peaks are observed in the FT-IR spectra of the electro-sprayed NPs that cannot be identified in the pure compounds. These results demonstrate the successful inclusion of NAT and CLZ in the CTS/PLGA NPs without chemical bond formation.

### X-Ray Diffraction

The X-ray diffractograms of the individual components and the electro-sprayed NPs are presented in Figures 3C,D. Crystalline nature played an important role in the stability, bioavailability, and solubility of the delivery system (Govender, 2015). The definitive diffractograms of CTS were two high-intensity peaks (10.420°, 19.889°), and PLGA was a broad and low peak at around 19.669°. In addition, the sharp peaks at 20.637° and 12.493° clearly indicate the crystalline nature of the NAT and CLZ, respectively. However, the XRD pattern of the NAT/CLZ-loaded electro-sprayed NPs shows only weak peaks corresponding to CTS, NAT, and CLZ. This may be attributed to the masking of these characteristic signals by the semi-crystalline PLGA. Nevertheless, the results demonstrate that the NAT and CLZ were successfully loaded onto the electro-sprayed NPs. Moreover, according to previous work, the weak crystallinity of PLGA is helpful in providing the drug molecules with increased solubility and bioavailability (Bolla et al., 2019).

### Thermogravimetric Analysis

The thermal behaviors of various formulations are revealed by the TGA curves in Figures 3E,F. Here, each formulation exhibits a weight loss of 5–10% over the temperature range of 40–200°C due to the evaporation of water or solvent molecules. Subsequent weight loss is ascribed to the breakdown of the various components. Thus, between 280 and 380°C, the PLGA exhibits rapid and extreme loss of ultimately 90% of its initial weight. By contrast, the CTS exhibits a weight loss of just 61.07% over the temperature range of 280 to 350°C, thus indicating better thermal stability than that of the PLGA. This is attributed to the crystalline nature of the CTS. Similarly, the NAT exhibits an initial weight loss in the temperature range of 280-300°C, whereas the CLZ exhibits an initial weight loss in the range of 300–310°C. In addition, the drug-loaded electro-sprayed NPs exhibit similar thermal stabilities to those of the pure NAT and CLZ. Furthermore, compared with the precipitous weight loss of the pure PLGA, the electro-sprayed NPs began to degrade only slowly at 300°C, thus demonstrating higher thermal stability. This good thermal stability is beneficial for the increased shelf-life of the formulation under typical storage conditions.

### *In vitro* Drug Release

**FIGURE 4:**
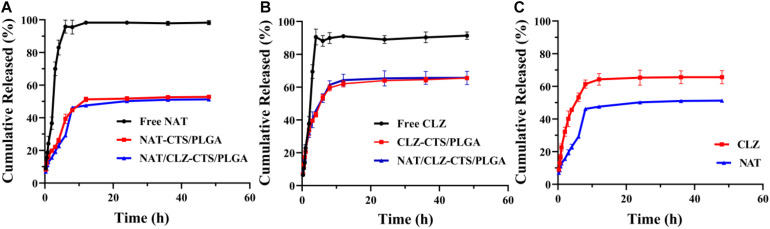
*In vitro* release study (0.2 M PBS, pH 7.4). **(A)** Profiles for NAT release from various formulations. **(B)** Profiles for CLZ release from various formulations. **(C)** Profiles for NAT and CLZ release from NAT/CLZ@CTS/PLGA NPs.

The release behavior of electro-sprayed NPs was studied in PBS (0.2 M, pH 7.4, 37°C), which simulated ocular circumstances with FK (Bhatta et al., 2012). The drug release curves of the various formulations are presented in Figure 4. Here, an almost 100% release of the NAT and CLZ is observed within 4 h from the start of the study. Moreover, the CLZ@CTS/PLGA NPs released 30% of the CLZ during the first 2 h, while the NAT/CLZ@CTS/PLGA NPs released 33% of the CLZ during this initial period. By contrast, the NAT@CTS/PLGA NPs released 15% of the NAT during the first 2 h, and the NAT/CLZ@CTS/PLGA NPs released 20% of the NAT during this time. After 48 h, the cumulative release of CLZ was 65.57 ± 0.92% and 65.64 ± 4.05% from the CLZ@CTS/PLGA NPs and the NAT/CLZ@CTS/PLGA NPs, respectively, while the cumulative amounts of NAT released from the CLZ@CTS/PLGA NPs and NAT/CLZ@CTS/PLGA NPs were 57.79 ± 0.18% and 51.28 ± 0.11%, respectively, at this time.

The mechanism of drug release from NPs involves diffusion, dissolution, and degradation of the polymer matrix (Jain et al., 2011). The incomplete release behavior may be related to the drug being embedded in the copolymer and drug would be further released after carrier degradation (Lee et al., 2010). Nevertheless, the initial burst release allows the drugs to reach an effective therapeutic concentration in the shortest possible time, which is beneficial for the treatment of FK. By contrast with the administration of the free drugs (NAT and CLZ), the sustained release from the core-shell NPs demonstrates that the developed NPs can be used as an important platform for continuous drug release. This characteristic is expected to prove very useful in providing long-lasting anti-inflammatory effects with low administration frequency (Luo et al., 2020).

### Hemolysis Assay

**FIGURE 5:**
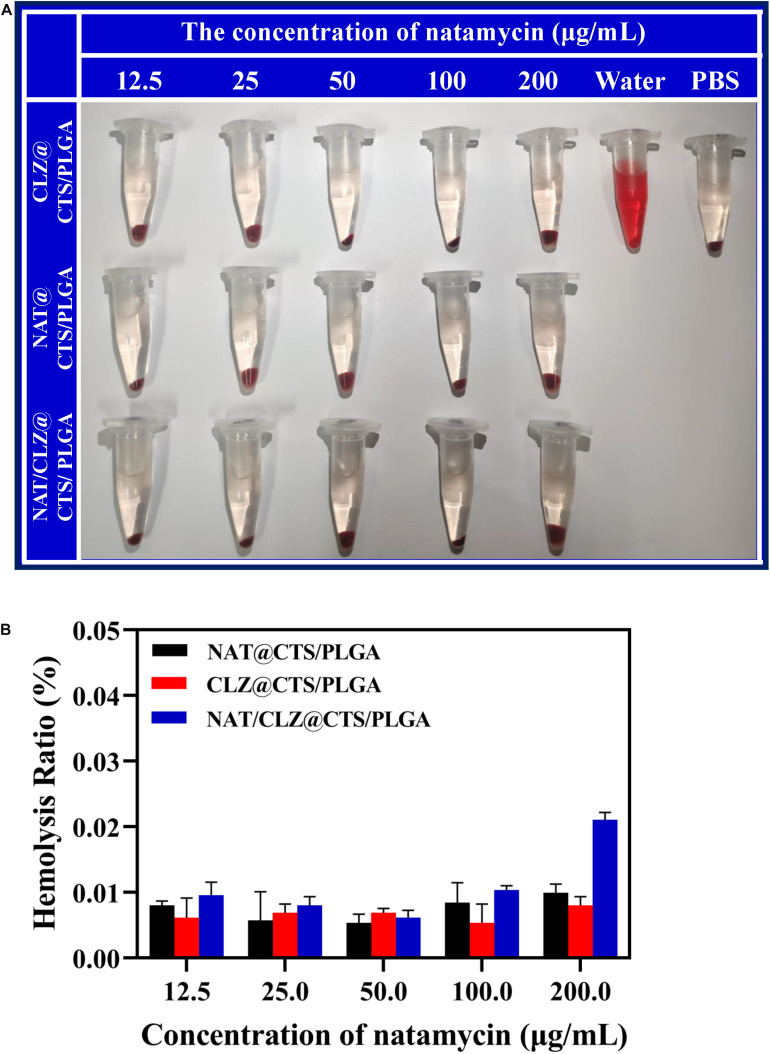
Hemolysis activity of various core-shell NPs. **(A)** The photograph of fresh rabbit blood incubated with different concentrations of various core-shell NPs. **(B)** Hemolysis ratio of different concentrations of various core-shell NPs.

Hemolysis assay was evaluated as a common *in vitro* method to the eye irritation test, which has been used to test medicinal excipients, such as cyclodextrin, for potential ocular irritation (Abdelkader et al., 2015). The hemolysis assay was performed for the various formulations according to the method described in section “Hemolysis Assay” (Figure 5). At all concentrations, the hemolysis ratio was found to be less than 1%, which is the critical safe hemolytic ratio for biomaterials according to ISO/TR 7406 (Li et al., 2012). These results provide a preliminary prediction that the NPs will not cause ocular tissue stimulation, cell lysis, or protein denaturation; however, further *in vivo* corneal irritation tests are needed to predict corneal stimulation (Nunes Alves et al., 2008).

### Antifungal Activity Analysis

**FIGURE 6:**
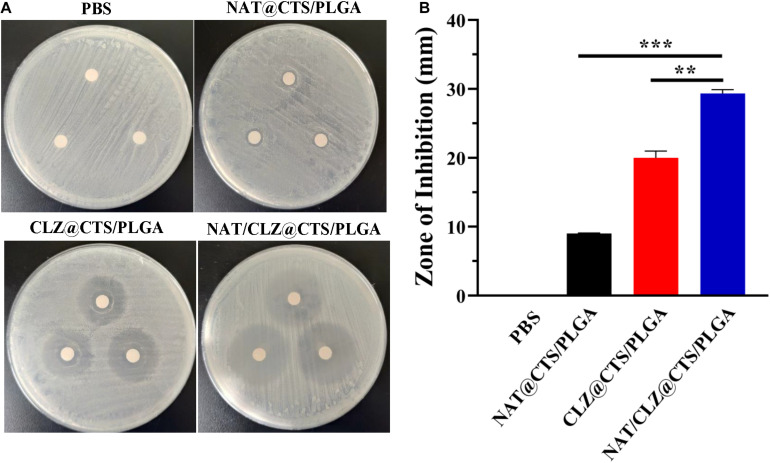
Antifungal activities against *C. albicans*. **(A)** The zone of *C. albicans* growth inhibition on the cultures of different formulations. **(B)** The zone of inhibition diameter graph. **P < 0.01, ***P < 0.0001.

The *in vitro* antifungal activity of the electro-sprayed NPs against *C. albicans* is indicated by the well diffusion bioassay results shown in Figure 6. The PBS group provides the negative control, and size of the ZOI reflects the capacity of the electro-sprayed NPs to inhibit the growth of *C. albicans* or, equivalently, the sensitivity of the organism to the agent (Owayss et al., 2020). The results indicate that after 48 h of incubation with *C. albicans*, the ZOI of NAT@CTS/PLGA NPs, CLZ@CTS/PLGA NPs, and NAT/CLZ@CTS/PLGA NPs were 9 ± 0.1, 20 ± 0.1, and 30 ± 0.06 mm, respectively. Thus, the NAT/CLZ@CTS/PLGA NPs provide significantly higher *in vitro C. albicans* inhibition than either the PBS, the NAT@CTS/PLGA NPs, or the CLZ@CTS/PLGA NPs. Furthermore, the antifungal activity of the NAT/CLZ@CTS/PLGA NPs is higher than that of the NAT-containing formulation that was previously synthesized by P. K. Shukla et al. (Bhatta et al., 2012). This result supports the present authors’ hypothesis that the combination of CLZ with NAT will have a synergistic antifungal effect and demonstrates the potential applicability of the developed NPs as to the treatment of FK.

### Ocular Irritation Studies

**FIGURE 7:**
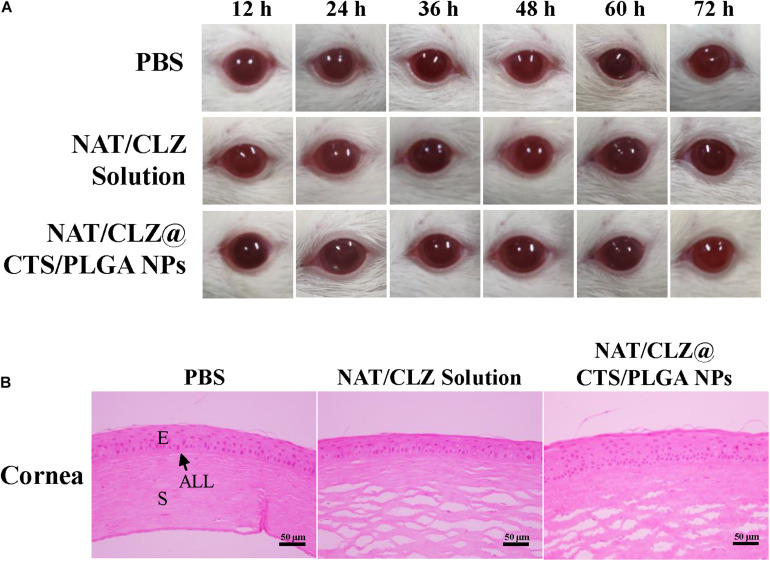
Ophthalmic irritation studies. **(A)** Ocular observation. **(B)** Histology analysis (H&E stain). The rats’ eyes were treated with PBS, NAT/CLZ solution, and NAT/CLZ@CTS/PLGA NP formulations for 12 consecutive hours (E, corneal epithelium; ALL, anterior limiting; S, corneal stroma. Scale bar = 50 μm).

To identify any damaging effects to the cornea (e.g., conjunctival congestion, corneal opacity, swelling, secretions, and redness) (Jain et al., 2011; Chan et al., 2020), the ocular irritancy test was performed according to the modified Draize test described in section “Ocular Irritation Studies,” and the results are presented in Figure 7A. Here, no irritation or damage to the cornea is evident at 12, 24, 36, 48, and 72 h from the last instilment. Hence, the corneal opacity grades of the various formulations are all zero. This macroscopic observation is supported by the results of H&E staining presented in Figure 7B, which reveal no obvious histological or structural changes in the cornea and no leukocytic infiltration. Thus, the absence of any signs of irritation or damage to the cornea further demonstrates the potential clinical application of the NAT/CLZ@CTS/PLGA NPs.

### Local Allergenic Tests

**FIGURE 8:**
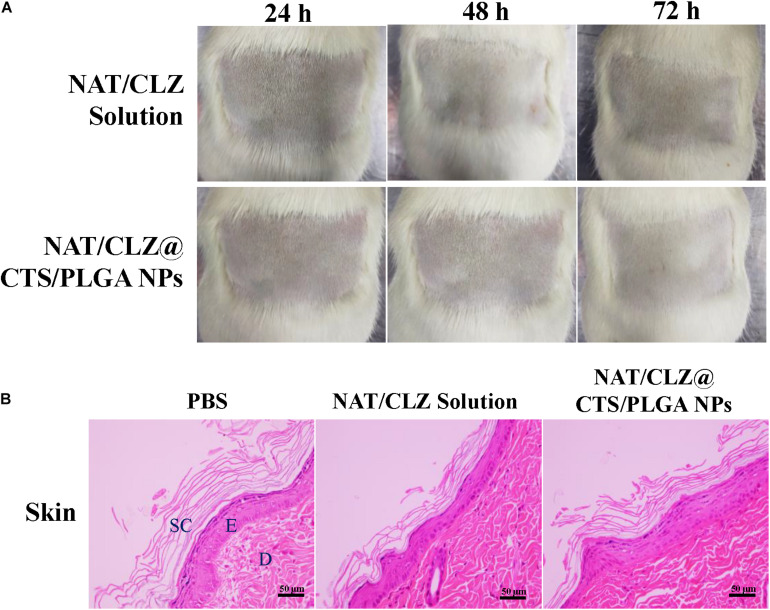
Local allergenic tests. **(A)** Skin observation. **(B)** Histology analysis (H&E stain). The skin of the exposed areas was excised from the animals at 72 h for histological studies (SC, stratum corneum; E, epidermis; D, dermis. Scale bar = 50 μm).

Local allergenic test was performed according to the procedure described in section “Local Allergenic Tests,” and the results are presented in Figure 8A. Here, in contrast to the observed effects in the PBS control group, the treatment groups exhibit no significant skin disruption. In particular, no skin dryness and no inflammatory skin reaction (which is the important parameter of skin barrier damage) are observed in the rats that were given the active formulation (Ahaghotu et al., 2005).

After H&E staining of the unexposed (negative control) skin and the exposed skin, the skin that was exposed to the NAT/CLZ@CTS/PLGA NPs and NAT/CLZ both exhibit intact epidermal and dermal layers without any disruption and no inflammatory cell, which is similar to that of the PBS groups (Figure 8B). Thus, the histological study demonstrates that the use of the NAT/CLZ@CTS/PLGA NPs as an anti-FK formulation would not cause local allergy to the rats. Moreover, since the selected test animal is known to be more sensitive than humans, this result provides strong support for the potential clinical application of the developed formulation (Valenta et al., 2018).

## Conclusion

A co-loaded drug delivery system incorporating NAT and CLZ along with the biocompatible CTS and the biodegradable PLGA was successfully fabricated herein via a one-step process using CO-ES technology. The electro-sprayed NPs exhibited a core-shell structure with a particle size range of 309–406 nm. In addition, the NAT and CLZ exhibited high EE values of 85.63 ± 0.04% and 89.61 ± 0.04%, respectively, when these drugs were co-loaded in the NAT/CLZ@CTS/PLGA NPs. Moreover, the electro-sprayed NPs were shown to provide a sustained drug release that could effectively reduce the necessary dosing frequency and improve the compliance of patients. The NAT/CLZ@CTS/PLGA NPs were also shown to exhibit excellent blood compatibility along with no significant corneal irritation *in vitro*, thus indicating their suitability for ocular delivery. In addition, the developed NPs exhibited significantly better antifungal activity as compared to that of the single drug-loaded NPs, thus indicating the good efficacy of the co-loaded NPs for the treatment of FK. Based on the *in vivo* ocular irritation study and local allergenic testing, the safety of the developed NPs was also confirmed. The present study suggests that the NAT/CLZ@CTS/PLGA NPs display excellent characteristics and can serve as a promising delivery system for the treatment of FK.

## Data Availability Statement

The original contributions presented in the study are included in the article/supplementary material, further inquiries can be directed to the corresponding authors.

## Ethics Statement

The animal study was reviewed and approved by the Animal Ethics Committee of Weifang Medical University.

## Author Contributions

All authors listed have made a substantial, direct and intellectual contribution to the work, and approved it for publication.

## Conflict of Interest

The authors declare that the research was conducted in the absence of any commercial or financial relationships that could be construed as a potential conflict of interest.
